# Changes in antioxidant status and DNA repair capacity are corroborated with molecular alterations in malignant thyroid tissue of patients with papillary thyroid cancer

**DOI:** 10.3389/fmolb.2023.1237548

**Published:** 2023-08-24

**Authors:** Zing Hong Eng, Azlina Abdul Aziz, Khoon Leong Ng, Sarni Mat Junit

**Affiliations:** ^1^ Department of Molecular Medicine, Faculty of Medicine, Universiti Malaya, Kuala Lumpur, Malaysia; ^2^ Department of Surgery, Faculty of Medicine, Universiti Malaya, Kuala Lumpur, Malaysia

**Keywords:** reactive oxygen species, oxidative DNA damage and repair, signalling pathways, papillary thyroid cancer, benign thyroid goitre, whole-exome sequencing

## Abstract

**Introduction:** Papillary thyroid cancer (PTC) accounts for approximately 80% of all thyroid cancer cases. The mechanism of PTC tumourigenesis is not fully understood, but oxidative imbalance is thought to play a role. To gain further insight, this study evaluated antioxidant status, DNA repair capacity and genetic alterations in individuals diagnosed with benign thyroid lesion in one lobe (BTG) and PTC lesion in another.

**Methods:** Individuals with coexisting BTG and PTC lesions in their thyroid lobes were included in this study. Reactive oxygen species (ROS) level, ABTS radical scavenging activity, ferric reducing antioxidant capacity, glutathione peroxidase and superoxide dismutase activities were measured in the thyroid tissue lysate. The expression of selected genes and proteins associated with oxidative stress defence and DNA repair were analysed through quantitative real-time PCR and Western blotting. Molecular alterations in genomic DNA were analysed through whole-exome sequencing and the potentially pathogenic driver genes filtered through Cancer-Related Analysis of Variants Toolkit (CRAVAT) analysis were subjected to pathway enrichment analysis using Metascape.

**Results:** Significantly higher ROS level was detected in the PTC compared to the BTG lesions. The PTC lesions had significantly higher expression of *GPX1*, *SOD2* and *OGG1* but significantly lower expression of *CAT* and *PRDX1* genes than the BTG lesions. Pathway enrichment analysis identified “regulation of MAPK cascade,” “positive regulation of ERK1 and ERK2 cascade” and “negative regulation of reactive oxygen species metabolic process” to be significantly enriched in the PTC lesions only. Four pathogenic genetic variants were identified in the PTC lesions; *BRAF* ^V600E^, *MAP2K7*-rs2145142862, *BCR*-rs372013175 and *CD24* NM_001291737.1:p.Gln23fs while *MAP3K9* and *G6PD* were among 11 genes that were mutated in both BTG and PTC lesions.

**Conclusion:** Our findings provided further insight into the connection between oxidative stress, DNA damage, and genetic changes associated with BTG-to-PTC transformation. The increased oxidative DNA damage due to the heightened ROS levels could have heralded the BTG-to-PTC transformation, potentially through mutations in the genes involved in the MAPK signalling pathway and stress-activated MAPK/JNK cascade. Further *in-vitro* functional analyses and studies involving a larger sample size would need to be carried out to validate the findings from this pilot study.

## 1 Introduction

Thyroid nodules are very common, with an occurrence rate of up to 70% in the general population if ultrasonography is performed (Dean and Gharib, 2008). While most of these nodules are benign, 4%–6.5% of them are malignant (Patel et al., 2020). The most common type of thyroid malignancy is papillary thyroid cancer (PTC), accounting for approximately 80% of all thyroid cancer diagnoses (Abdullah et al., 2019). Fine needle aspiration cytology (FNAC) is the primary diagnostic tool to determine malignancy status of the nodules. The cytology findings are classified according to the Bethesda System for Reporting Thyroid Cytopathology (TBSRTC) framework (Renuka et al., 2012). Indeterminate malignancy status is typically confirmed through histopathological examination (HPE) of thyroid tissue samples obtained from a partial or total thyroidectomy (Paulson et al., 2019).

Molecular changes including *RET* chromosomal rearrangement, *BRAF* and *RAS* genes point mutations that lead to oncogenic activation of mitogen-activated protein kinase (MAPK) signalling pathway are frequently activated in PTC, leading to dysregulation of cell proliferation and survival (Abdullah et al., 2019). However, the exact molecular mechanism responsible for the onset of PTC malignant transformation is not fully known. While radiation exposure has been suggested as a possible factor (Saad et al., 2006), studies have also linked oxidative stress to the pathogenesis of PTC (Ameziane-El-Hassani et al., 2010).

Reactive oxygen species (ROS) is a group of oxygen-containing molecules produced as by-products of cellular metabolic processes (Reczek and Chandel, 2017). Under normal physiological conditions, the balance between the production and elimination of ROS within cells is maintained through various regulatory mechanisms, including the action of antioxidant enzymes (Zhang et al., 2021). Key antioxidant enzymes that are involved in ROS neutralisation include superoxide dismutase (SOD), glutathione peroxidase (GPx) and catalase (CAT). Furthermore, peroxiredoxins (PRDXs), particularly PRDX1 and PRDX6, have been implicated in PTC tumourigenesis, highlighting their significance within the antioxidant enzyme system (Nicolussi et al., 2014). However, when there is an imbalance between the production and elimination of ROS, the result can be oxidative stress, leading to damage to cellular macromolecules including DNA (Ramli et al., 2017). The DNA damage is usually repaired through various DNA repair mechanisms including the base excision repair (BER) assisted by the primary enzyme, 8-oxoguanine DNA glycosylase (OGG1) (Chatterjee and Walker, 2017).

Due to the participation of hydrogen peroxide (H_2_O_2_) in thyroid hormone synthesis, the linkage between oxidative stress and thyroid tumourigenesis has raised many concerns. In this context, the present study evaluated the antioxidant status, DNA repair capacity and genetic alterations in individuals who exhibit benign thyroid lesion in one lobe (BTG) and malignant PTC in another lobe, with the aim to further understand the molecular events that may drive the development and progression of malignant PTC from benign BTG lesions.

## 2 Materials and methods

### 2.1 Subjects

This study was approved by the Universiti Malaya Medical Centre (UMMC)’s Medical Research Ethics Committee (MREC ID NO: 2019619-7540) in accordance with the ICH GCP guidelines and the Declaration of Helsinki. Written informed consent was obtained from all patients before the study was carried out.

Six patients who had malignant thyroid lesion in one lobe (PTC) and BTG in another, were included in the study. The malignancy status of the thyroid lobes of the patients was first evaluated through FNAC, which was then confirmed through HPE. Thyroid specimens from both the benign and malignant thyroid lobes of each individual were submerged in AllProtect tissue reagent (Qiagen, Hilden, Germany) at the time of retrieval and were then stored at −80°C.

### 2.2 Tissue lysate preparation for biochemical analysis

Thyroid tissue specimens from the BTG and PTC lesions were rinsed with phosphate buffered saline (PBS) (1×). Twenty milligrams of thyroid tissue samples were homogenised in 200 µL of cold PBS (1×). The tissue lysate was then centrifuged at 5,000 × g for 5 min at 4°C. The supernatant was then stored at −80°C until further analyses.

### 2.3 ROS level detection

Dichlorofluorescein diacetate (DCFH-DA) solution (20 µM) was added to 5 µL tissue lysate sample. The mixture was incubated for 30 min at room temperature and the fluorescence reading was taken with the excitation and emission wavelengths set as 485 nm and 530 nm, respectively. The fluorescence intensities were then standardised to the protein content. All reactions were done in triplicate.

### 2.4 2,2’-azino-bis(3-ethylbenzothiazoline-6-sulphonic acid) (ABTS) radical scavenging activity

ABTS radical scavenging activity stock solution was prepared by incubating 7 mM ABTS and 2.45 mM potassium persulphate at room temperature for 16 h in the dark. The solution was then diluted with methanol to obtain a working solution with an absorbance of 0.70 ± 0.02 at 415 nm. Four hundred microlitres of ABTS working solution were then added into four microlitres of tissue lysate samples. After a 10-min incubation period in the dark, the mixture was centrifuged at 1925 × g for 1 minute. The absorbance of the supernatant was then read at 415 nm. Trolox was used as standard, and the results were expressed as Trolox equivalent antioxidant capacity (TEAC). The analyses were done in triplicate.

### 2.5 Ferric reducing antioxidant power (FRAP) assay

The FRAP working reagent was prepared by mixing 10 parts of acetate buffer (300 mM, pH 3.6), one part of 2,4,6-tri (2-pyridyl)-S-triazine solution (10 mM) and one part of 20 mM FeCl_3_·6H_2_O in 40 mM HCl. The working reagent was preheated to 37°C before assay. Two hundred microlitres of the working reagent were then added into 2 µL of tissue lysate sample, the absorbance of the reaction mixture was then measured at 595 nm after 30 min of incubation at room temperature. Iron (II) sulphate solution (0–2000 μmol/L) was used to construct a calibration curve. Analyses were performed in triplicate and the result is denoted as nanomole Fe^2+^ per milligram of protein (nmol Fe^2+^/mg protein).

### 2.6 Glutathione peroxidase (GPx) activity

GPx activity in the tissue lysate samples were measured using the Glutathione Peroxidase Assay Kit (Cayman Chemical Company, Michigan, United States) according to the manufacturer’s protocol. The depletion of cumene peroxide in the reaction mixture was measured at 340 nm for 5 min with the interval of 1 minute. The GPx activity of the tissue lysate was expressed as nmol per minute in one mL of tissue lysate (nmol/min/mL). All reactions were performed in triplicate.

### 2.7 Superoxide dismutase (SOD) activity

SOD activity in the tissue lysate samples were performed using the Superoxide Dismutase Assay Kit (Cayman Chemical Company, Michigan, United States). The absorbance of the reaction mixture was read at 450 nm using a plate reader after a 30-min incubation. A serial dilution of SOD standards was used to construct the standard curve and the SOD activity in the tissue lysate was normalised against the linearised rate of the SOD standard. The SOD activity in tissue lysate sample was expressed as unit per millilitre sample (U/mL). All reactions were done in triplicate.

### 2.8 Genomic DNA, RNA and protein purification

After homogenising 30 mg of thyroid tissue in 600 µL of Buffer RLT pre-added with β-mercaptoethanol using TissueRuptor (Qiagen, Hilden, Germany), genomic DNA (gDNA), RNA and protein were extracted from the tissue lysate using Qiagen AllPrep DNA/RNA/Protein Mini Kit (Qiagen, Hilden, Germany) according to the manufacturer’s protocol. The concentration and purity of the extracted gDNA were determined using Qubit dsDNA BR Assay kit (Life Technologies, Darmstadt, Germany) on Qubit 2.0 Fluorometer, and by Thermo Scientific NanoDrop™ 2000c Spectrophotometer (Thermo Fisher Scientific, Massachusetts, United States), respectively. The yield and purity of the nucleic acids were estimated using Nanodrop 2000c Spectrophotometer. The gDNA and RNA integrity tests were performed by 1% agarose gel electrophoresis.

### 2.9 Gene expression analysis of *OGG1, PRDX1, PRDX6, CAT, GPX1, SOD1* and *SOD2*

The expression of *8-oxoguanine DNA glycosylase* (*OGG1*), *peroxiredoxin 1* (*PRDX1)*, *peroxiredoxin 6* (*PRDX6*), *catalase* (*CAT*), *glutathione peroxidase 1* (*GPX1*), *superoxide dismutase 1* (*SOD1*) *and superoxide dismutase 2* (*SOD2*) genes were compared between the benign and malignant lesions of each of the six patients. Four hundred micrograms of the purified RNA were converted to complementary DNA (cDNA) using qPCRBIO cDNA Synthesis Kit (PCR Biosystems, London, United Kingdom). Quantitative real-time PCR (qRT-PCR) was performed using Applied Biosystems StepOnePlus Real-Time PCR System (Thermo Fisher Scientific, Massachusetts, United States). In a final volume of 20 µL qRT-PCR reaction mixture, the following components were included; 50 ng of cDNA, forward and reverse primers 400 nM each, 10 µL of qPCRBIO SyGreen Mix (2×) (PCR Biosystems, London, United Kingdom), and nuclease-free water. The reaction mixture was first incubated at 95°C for 2 minutes for polymerase activation, followed by 40 amplification cycles of 5 seconds of denaturation at 95°C and 20 s of annealing/extension process at 60°C. Melting curve analysis was performed for each primer pair to confirm single discrete species of amplification product. All qRT-PCR experiments were performed in triplicate. The gene expressions of the targeted genes were standardised against 18S ribosomal RNA. The primers used in the study are included in Supplementary Table S1.

### 2.10 Western blot analysis of OGG1 and PRDX6

After protein pellet reconstitution in 5% (w/v) of SDS solution, protein content was quantitated using Pierce^™^ BCA Protein Assay Kit (Thermo Fisher Scientific, Masshachusetts, United States). Twenty micrograms of protein from 12 samples (six benign and six malignant lesions from the six patients) were first separated in a 12.5% sodium dodecyl sulphate polyacrylamide gel. The proteins were then transferred onto a nitrocellulose membrane. The OGG1 and PRDX6 expressions were normalised to the expression of β-actin. The following antibodies were used; OGG1 Rabbit pAb antibody (ABclonal, Cat # A1384, 1:1,000), PRDX6 Polyclonal Antibody (Elabscience, Cat # E-AB-60566, 1:1,000) and Anti-beta Actin antibody (Abcam, Cat # ab8227, 1:2,000). The presence of the targeted proteins was detected using Invitrogen™ WesternDot™ 625 Goat Anti-Rabbit Western blot Kit (Thermo Fisher Scientific, Massachusetts, United States). The membrane was then visualised using ChemicDoc XRS + System (Bio-Rad Laboratories, California, United States) and analysed using ImageJ software.

### 2.11 Whole-exome sequencing

gDNA of the malignant and concurrent benign lesions from three patients were sent to BGI Biotechnology Company (Shenzhen, China) for whole-exome sequencing (WES). The gDNA was randomly fragmented to about 150 bp to 250 bp prior to ligation with adapters for amplification and sequencing. After ligation-mediated PCR, the purified products were then hybridised to the Agilent SureSelect Human All Exon V6 exome array for enrichment. The captured products were then circularised and rolling circle amplification (RCA) was performed to produce DNA Nanoballs. The captured libraries were sequenced using the BGISEQ-500 sequencing platform. The WES data in FASTQ format were then aligned to GRCh37 human reference genome using Burrows-Wheeler Aligner (BWA) software. Genomic variations were detected by Genome Analysis Toolkit (GATK) (v3.6). The single nucleotide variants (SNVs) and small insertions and deletions (InDels) were annotated using the SnpEff program (http://snpeff.sourceforge.net/). Variant call format (VCF) files for each gDNA samples were generated after variant annotation process.

### 2.12 Identification of driver mutations

The VCF files were subjected to the Cancer-Related Analysis of Variants toolkit version 4.3 (CRAVAT 4.3) (http://hg19.cravat.us/CRAVAT/) to filter out false positive variants and identify potential driver somatic mutations (Douville et al., 2013). Three analysis tools were chosen in CRAVAT, which were Cancer-specific High-throughput Annotation of Somatic Mutations 3.0 (CHASM 3.0), Variant Effect Scoring Tool 3.0 (VEST 3.0) and SNVGet. Particularly for CHASM 3.0, “Thyroid” was chosen as the disease type. The CRAVAT prioritised SNVs and InDels with the following criteria: (a) MAF ≥0.01 in the 1,000 Genome Project, the NHLBI-ESP6500 and the ExAC control databases; (b) synonymous variants; (c) non-frameshift InDel variants; were excluded. Missense variants with the *p*-value of ≥0.05 in both CHASM and VEST predicting tools were excluded. The frameshift InDels, splice site variants, stop gain variants, stop loss variants with the *p*-value of ≥0.05 in VEST tool were excluded. The list of genes that contain the filtered variants were used for the subsequent pathway and process enrichment analysis.

### 2.13 Pathway and process enrichment analysis

Pathway and process enrichment analysis was carried out using Metascape (https://metascape.org/) (Zhou et al., 2019), where the CRAVAT-prioritised genes were used as the input gene sets. The enrichment analysis was then carried out based on the Gene Ontology (GO) Biological Processes database. All genes in the genome were used as the enrichment background. The parameters set for the enrichment analysis were as follows; a minimum overlap of three gene counts, minimum enrichment factor of 1.5 with a *p*-value of <0.01. The significantly enriched terms were then grouped into clusters based on their membership similarities.

### 2.14 *In-silico* functional analysis of the PTC-specific pathogenic variants

Three *in silico* prediction tools were used to assess the functional effects of the variants that were exclusively identified in the PTC lesions through CRAVAT filtering process, which are PolyPhen-2 (http://genetics.bwh.harvard.edu/pph2/), Sorting Intolerant From Tolerant (SIFT, https://sift.bii.a-star.edu.sg/index.html) and Mutation Taster (https://www.mutationtaster.org/). The WES sequencing results were validated by *BRAF* ^V600E^ mutation screening using Sanger sequencing. Primers targeting the *BRAF* mutation were designed using Primer3 (https://primer3.ut.ee/): Forward: 5’-CTC​TTC​ATA​ATG​CTT​GCT​CTG​ATA​G-3’; Reverse: 5’-CCT​CAA​TTC​TTA​CCA​TCC​AC-3’.

### 2.15 Statistical analysis

All data of oxidative stress biomarkers and antioxidant analyses, gene expression and protein expression analyses were expressed as mean ± SEM. Statistical analysis was performed using GraphPad Prism 8 (GraphPad Software Inc., California, United States). Student’s *t*-test was used to compare the mean differences of oxidative stress biomarkers and antioxidative response, gene expression and protein expression analyses, between BTG lesions and PTC lesions. *p*-values less than 0.05 were considered significant.

## 3 Results

### 3.1 Oxidative stress and antioxidant response in tissue lysates

**FIGURE 1:**
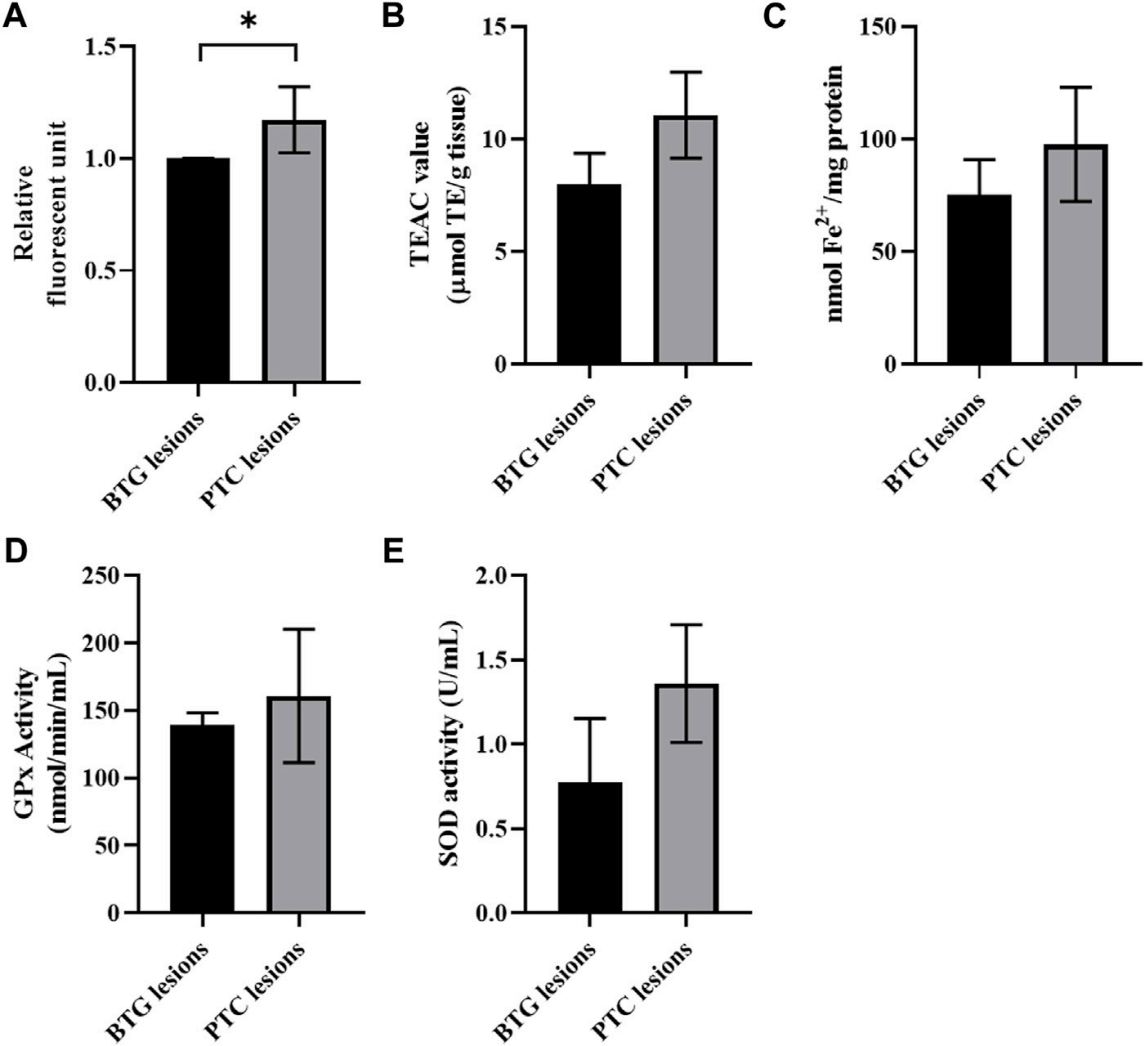
Oxidative stress and antioxidant response in tissue lysate samples from benign and malignant lesion. **(A)** ROS detection analysis; **(B)** ABTS radical scavenging activity; **(C)** ferric reducing antioxidant power (FRAP) assay; **(D)** glutathione peroxidase activity; **(E)** superoxide dismutase (SOD) activity. * indicates significant difference (*p* < 0.05) between benign and malignant lesions.

Figures 1A–E show the ROS level, antioxidant capacities and antioxidant enzyme activities in thyroid tissue lysate samples from benign and malignant lesions. Tissue lysate from the PTC lesions showed significantly higher ROS level compared to the BTG lesions (*p* < 0.05) (Figure 1A). The antioxidant capacity measured using ABTS radical scavenging activity and FRAP assay showed higher antioxidant activities in the PTC lesions, albeit not significantly (Figures 1B, C). Meanwhile, the GPx and SOD enzymatic activities in the PTC lesions were also found to be higher compared to the BTG lesions, but not statistically significant (*p* > 0.05) (Figures 1D, E).

### 3.2 Differential gene expression of *OGG1*, *CAT*, *GPX1*, *SOD2* and *PRDX1* in benign and malignant lesions

**FIGURE 2:**
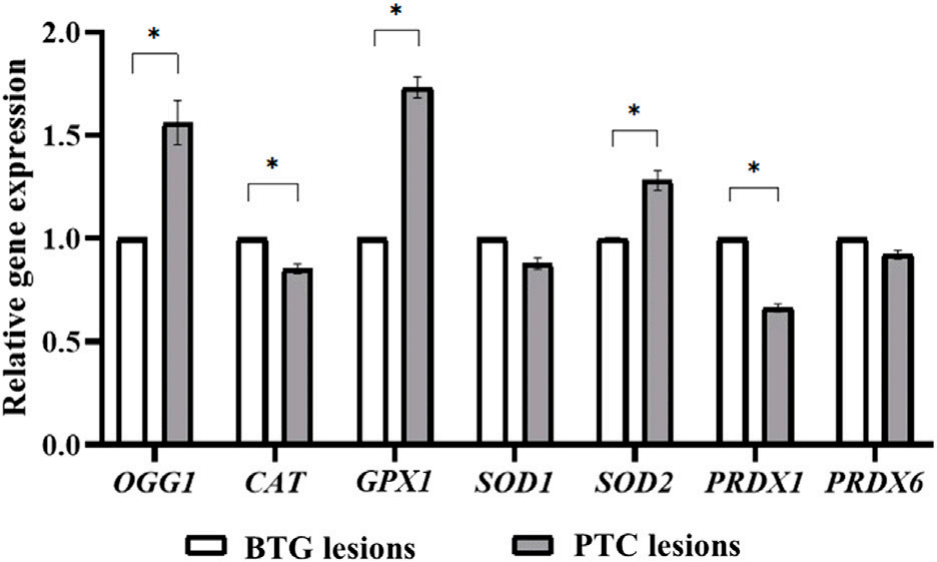
Gene expression analysis of *OGG1*, *CAT*, *GPX1*, *SOD1*, *SOD2*, *PRDX1* and *PRDX6* through qRT-PCR. * indicates significant difference (*p* < 0.05) between the gene expression in benign and malignant lesions.

The relative expressions of the *OGG1*, *CAT*, *GPX1*, *SOD1*, *SOD2*, *PRDX1* and *PRDX6* genes in the benign and malignant lesions are shown in Figure 2. The expression of *OGG1*, *GPX1* and *SOD2* in the PTC lesions was significantly higher than in the BTG lesions (*p* < 0.05). On the other hand, the expression of *CAT* and *PRDX1* genes was significantly lower in the PTC lesions compared to the BTG lesions. The expression of *SOD1* and *PRDX6* genes showed no significant difference between the malignant and the benign lesions.

### 3.3 Upregulated OGG1 protein expression in malignant PTC lesions

**FIGURE 3:**
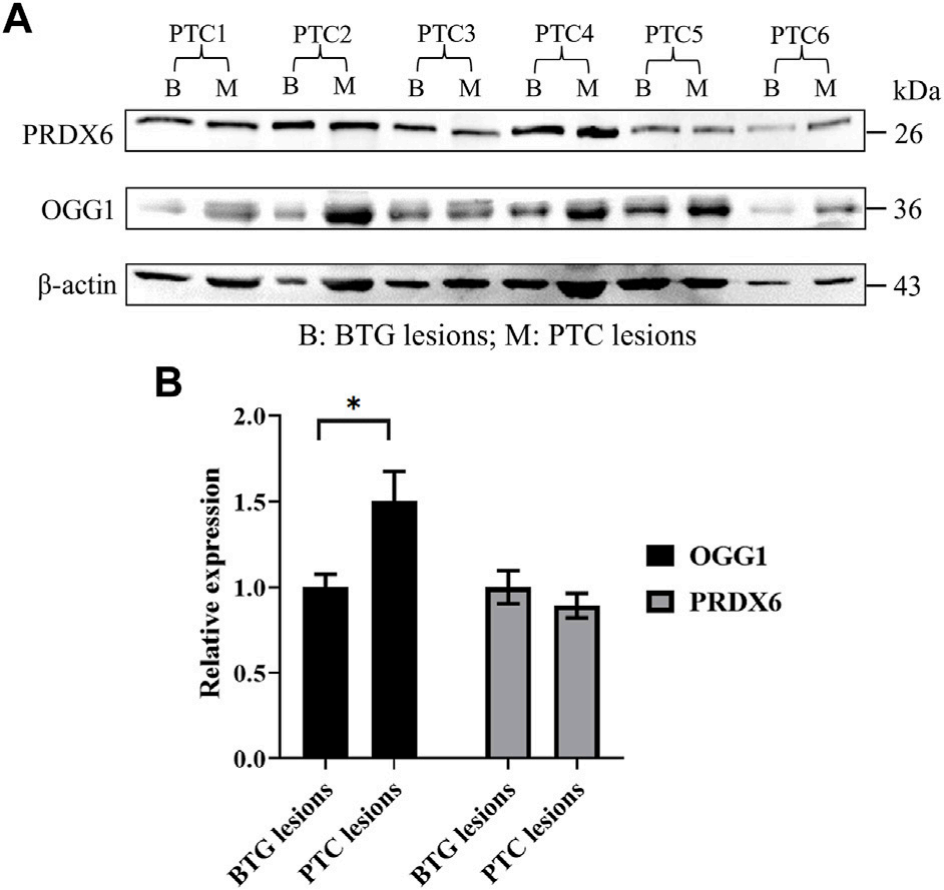
Protein expression analysis of OGG1 and PRDX6 in the BTG and PTC lesions from six PTC patients through Western blotting. **(A)** Western blot gel image of β-actin, OGG1 and PRDX6 in BTG lesions and PTC lesions of the patients. **(B)** The relative expression of OGG1 and PRDX6 in PTC lesions against BTG lesions after normalisation to the β-actin expression. * indicates significant difference (*p* < 0.05) between the gene expression in benign and malignant lesions.

Western blot analysis demonstrated the presence of OGG1 and PRDX6 proteins represented by the 36 kDa and 26 kDa bands respectively (Figure 3A), in the benign and malignant lesions of the patients. Densitometric evaluation of the blots using ImageJ showed approximately 1.5-fold higher expression of OGG1 protein in the PTC lesions compared to those of the BTG lesions (*p* < 0.05) (Figure 3B). No difference in the expression of PRDX6 was detected between the benign and malignant lesions in all patients.

### 3.4 WES analysis of the BTG and PTC lesions

Variant calling was performed on 60.46 Mb target region that were captured in this study. Total clean reads per sample were aligned to the human reference genome using Burrows-Wheeler Aligner (BWA). On average, 99.82% were mapped successfully. The duplicate reads were removed, resulting in the average of 118.62 Mb effective reads. Of the total effective bases, 47.03% were mapped on target regions. On average per sequencing individual, 99.85% of targeted bases were covered by at least 1× coverage and 94.80% of the targeted bases had at least 20× coverage. The summarised statistic of alignment is presented in Supplementary Table S2.

### 3.5 Identification of coding variants

WES identified a total of 36,909 SNVs and 1,023 InDels in the coding regions of the gDNA samples from three BTG lesions and three PTC lesions (Table 1). The average of the transition to transversion ratio (Ti/Tv) of the coding SNVs was found to be 2.285. Of these SNVs, 18,361 were synonymous, 18,129 were missense, 169 were stop gain, 46 were stop loss, 42 were start loss and 162 were splice site. Frameshift variant was the commonest InDels, where 473 frameshift variants were annotated. There were 395 non-frameshift, 148 splice site, four start loss, two stop loss and one stop gain InDels identified in our patient cohorts.

**TABLE 1:** Coding SNVs and InDels identified in malignant and benign lesions of the three patients.

Sample	PTC1		PTC2		PTC3		Overall
	BTG	PTC	BTG	PTC	BTG	PTC	
Coding single nucleotide variants							
Synonymous	11,440	11,367	11,388	11,383	11,455	11,476	18,361
Missense	10,870	10,725	10,808	10,842	10,888	10,941	18,129
Stop gain	96	93	81	83	80	85	169
Stop loss	33	32	36	36	32	31	46
Start loss	26	28	23	23	20	20	42
Splicing	90	93	101	101	88	90	162
Ti/Tv	2.29	2.28	2.29	2.29	2.28	2.28	2.284
Coding InDel variants							
Frameshift	291	299	301	301	269	269	473
Non-frameshift Insertion	78	85	91	91	68	69	143
Non-frameshift Deletion	139	141	132	123	117	116	252
Stop gain	1	1	1	1	1	1	1
Stop loss	0	0	0	0	2	1	2
Start loss	2	2	3	2	2	2	4
Splicing	86	95	101	98	86	85	148

Ti/Tv, transition to transversion ratio; BTG, benign thyroid goitre lesion; PTC, papillary thyroid cancer lesion.

### 3.6 Potential pathogenic driver variants identified in BTG and PTC lesions

**FIGURE 4:**
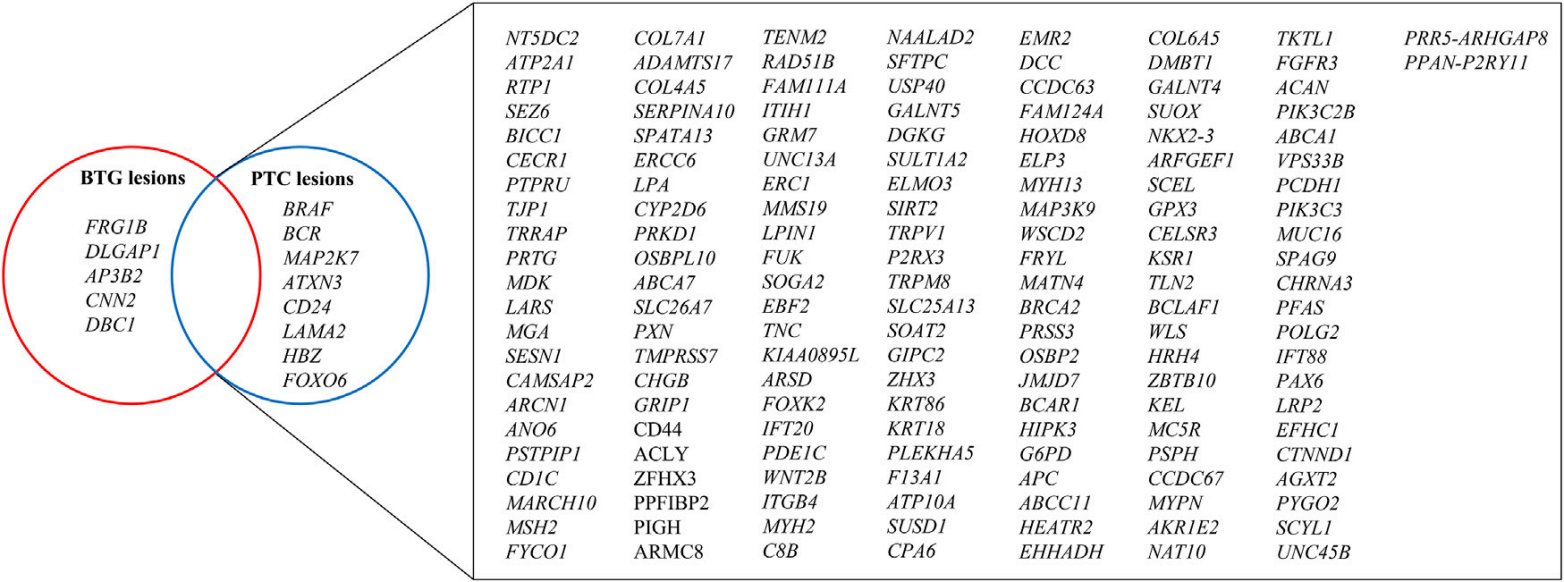
Venn diagram illustration of the genes contained filtered variants after CRAVAT analysis in BTG and PTC lesions. Nonsynonymous SNVs with *p*-values <0.05 in both CHASM and VEST algorithms were prioritised, whereby InDels and splice site variants with *p*-values <0.05 in VEST algorithm were prioritised. The genes with these retained variants were then used for pathway and process enrichment analysis.

After the CRAVAT analysis of the VCF files of the three BTG and three PTC lesions, a total of 169 genes were found to harbour the variants that conformed to the inclusion criteria (Figure 4). Among the 169 genes, 156 genes were shared between the BTG and PTC lesions, including *CD44*, *MAP3K9*, *MYH2* and *G6PD*. There were eight genes that were only identified in the PTC lesions, namely *BRAF*, *BCR*, *MAP2K7*, *ATXN3*, *CD24*, *LAMA2*, *HBZ* and *FOXO6*. In contrast, *FRG1B*, *DCGAP1*, *AP3B2*, *CNN2* and *DBC1* were found to be specific to BTG lesions.

### 3.7 Pathway and process enrichment analysis

**FIGURE 5:**
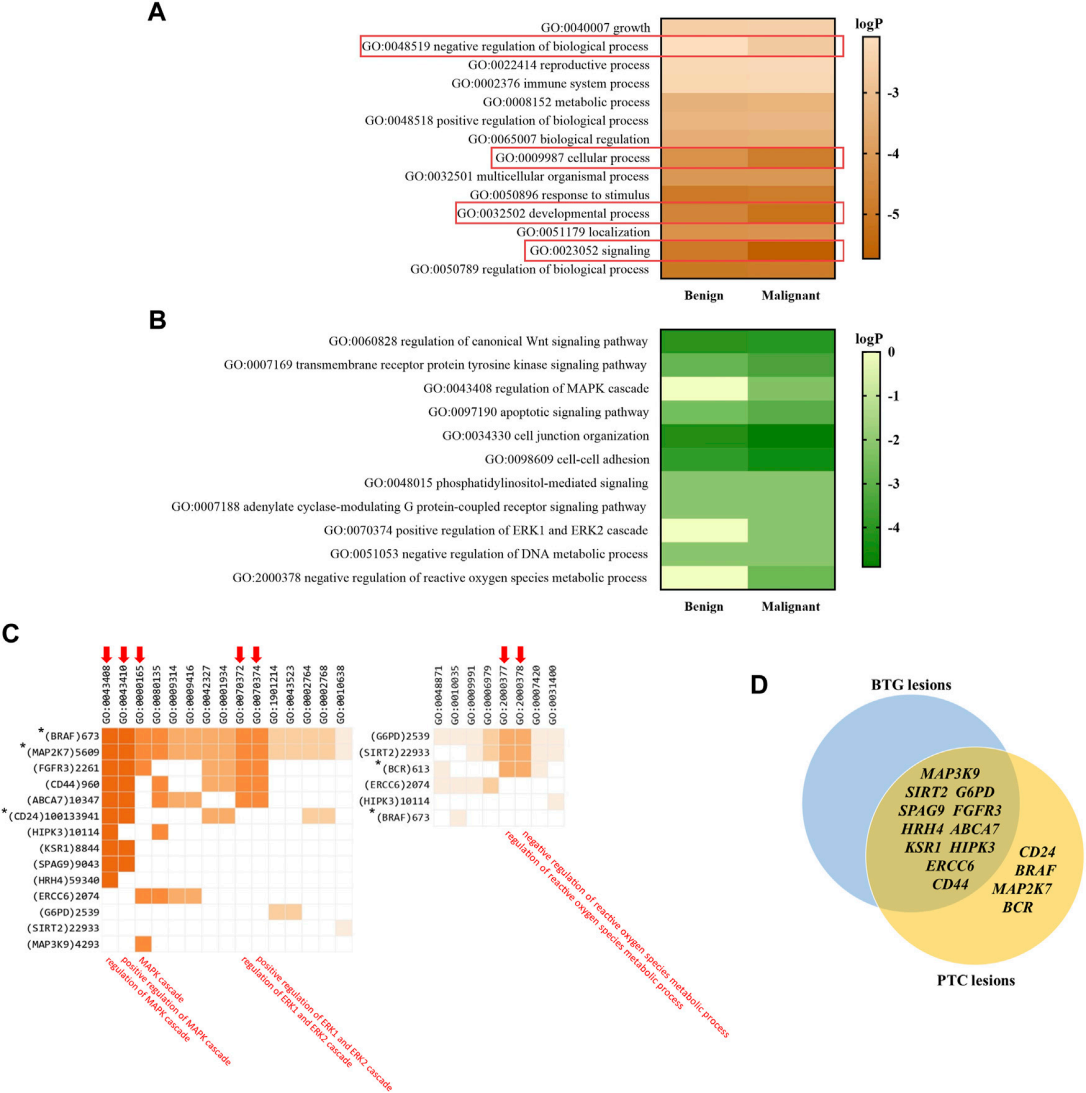
Gene enrichment analysis showing the top enriched pathways based on the GO ontology database. **(A)** The top enriched GO parental terms in benign and malignant lesions. The differentially enriched GO parental terms between benign and malignant lesions are red line bordered. **(B)** The top enriched GO terms within the four differentially enriched parental terms. **(C)** The genes involved in GO:0043408, GO:0070374 and GO:2000378. The three ontology terms and their closely related terms are indicated with arrows. * indicates the CRAVAT stratified driver genes that are presented in PTC lesions only. Ontology term with *p*-value <0.01 (LogP < −2) was considered as significant. **(D)** Venn diagram illustrates the distribution of the potential driver genes stratified by CRAVAT that involve in MAPK signalling cascade and its regulation, as well as regulation of ROS metabolic process, between BTG lesions and PTC lesions.

The top enriched GO parental terms are shown in Figure 5A, of which four of them were found to be differentially enriched in the benign and malignant lesion indicated by the rectangles; GO:0048519 (negative regulation of biological process), GO:0009987 (cellular process), GO:0032502 (developmental process), and GO:0023052 (signaling). The four enriched GO parental terms were refined into their daughter GO terms as shown in Figure 5B. GO:0060828 (regulation of canonical Wnt signaling pathway), GO:0007169 (transmembrane receptor protein kinase signaling pathway), GO:0043408 (regulation of MAPK cascade), GO:0097190 (apoptotic signaling pathway), GO:0034330 (cell junction organization), GO:0098609 (cell-cell adhesion), GO:0048015 (phosphatidylinositol-mediated signaling), GO:0007188 (adenylate cyclase-modulating G protein-coupled receptor signaling pathway), GO:0070374 (positive regulation of ERK1 and ERK2 cascade), GO:0051053 (negative regulation of DNA metabolic process) and GO:2000378 (negative regulation of reactive oxygen species metabolic process) were among the top significantly enriched pathways between the two groups. Particularly, GO:0043408, GO:0070374 and GO:2000378 were found to be significantly enriched in PTC lesions only. Figure 5C shows the genes that are involved in the enriched GO:0043408, GO:0070374 and GO:2000378 biological processes. The genes, namely *BRAF*, *MAP2K7*, *FGFR3*, *CD44*, *ABCA7*, *CD24*, *HIPK3*, *KSR1*, *SPAG9*, *HRH4*, *ERCC6* and *MAP3K9*, were found to be involved in the “regulation of MAPK cascade” and its related terms: “positive regulation of MAPK cascade” and “MAPK cascade”. Five out of the 12 genes, *BRAF*, *MAP2K7*, *FGFR3*, *CD44* and *ABCA7* were also found to be related to “positive regulation of ERK1 and ERK2 cascade”. On the other hand, *G6PD*, *SIRT2* and *BCR* were found to be associated with “negative regulation of reactive oxygen species metabolism process”. Figure 5D illustrates the contrasting distribution of genes related to MAPK signalling cascade and its regulation, as well as regulation of ROS metabolic process, between BTG lesions and PTC lesions. Among the 15 genes, *SPAG9*, *SIRT2*, *G6PD*, *CD44*, *FGFR3*, *HRH4*, *ABCA7*, *KSR1*, *HIPK3*, *ERCC6* and *MAP3K9* were shared between BTG lesions and PTC lesions. It is noteworthy that *BRAF*, *MAP2K7*, *CD24*, and *BCR* were predicted to be the potential driver genes and were only found in the PTC lesions. The results of the pathway enrichment analysis can be found in detail in Supplementary Table S3.

### 3.8 Potential driver mutations identified in PTC lesions and *in silico* functional analysis

**FIGURE 6:**
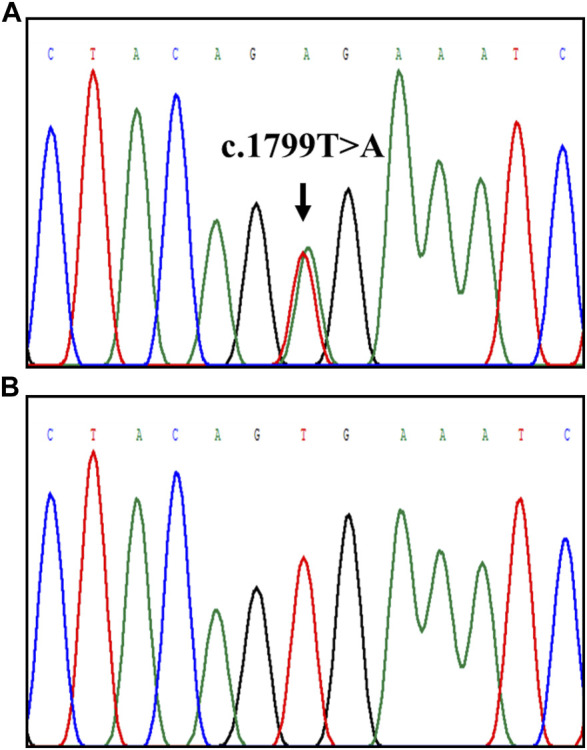
Detection of *BRAF-*rs113488022 (*BRAF* ^V600E^) through Sanger sequencing. The position of the c.1799T>A mutation is indicated by the arrow: **(A)** mutant *BRAF* ^V600E^ and **(B)** wildtype.

Four variants were identified to be potential driver mutations and were found exclusively in PTC lesions; *BRAF* NM_004333.4 c.1799T>A p.Val600Glu (*BRAF*-rs113488022), NM_001297555.1 c.880G>C p.Ala294Pro (*MAP2K7*-rs2145142862), *BCR* NM_004327.3 c.3275_3278dupCCGG p.Val1094fs (*BCR*-rs372013175) and *CD24* NM_001291737.1 c.67_68insTTTAATTTTATATGAGAGTACATGGAGGTAGCTGTGATGTGGAAATGTATCCATGTAACTTTTTATGTATTTT p.Gln23fs (Table 2). The two missense mutations (*BRAF*-rs113488022 and *MAP2K7*-rs2145142862) were predicted to be deleterious by PolyPhen-2, SIFT and Mutation Taster. Mutation Taster predicted that the *BCR*-rs372013175 to be a “Disease causing” variant. *BRAF*-rs113488022 validation using PCR-direct DNA sequencing is shown in Figure 6.

**TABLE 2:** Deleterious variants identified in the PTC lesions.

Chromosomal location (GRCh37)	Gene	Variant type	Transcript	HGVS.c	HGVS.p	dbSNP ID	*In-silico* functional analysis of the variant effect		
							PolyPhen-2 prediction	SIFT prediction	Mutation Taster
Chr7:140453136	*BRAF*	missense	NM_004333.4	c.1799T>A	p.Val600Glu	rs113488022	Damaging	Deleterious	Disease causing
Chr19:7976021	*MAP2K7*	missense	NM_001297555.1	c.880G>C	p.Ala294Pro	rs2145142862	Damaging	Deleterious	Disease causing
Chr22:23653975	*BCR*	frameshift	NM_004327.3	c.3275_3278dupCCGG	p.Val1094fs	rs372013175			Disease causing
ChrY:21154528	*CD24*	frameshift	NM_001291737.1	c.67_68insTTTAATTTTATATGAGAGTACATGGAGGTAGCTGTGATGTGGAAATGTATCCATGTAACTTTTTATGTATTTT	p.Gln23fs	N/A			

## 4 Discussion

Completion of thyroidectomy involves the removal of the contralateral thyroid lobe when the HPE result of the ipsilateral lobe reveals cancerous findings (Rosário et al., 2004). However, the histopathologic condition of the contralateral lobe is not always malignant. This study included six patients who exhibited PTC lesions in only one of the two thyroid lobes and BTG lesions in the opposite lobe. Earlier studies had reported significantly higher oxidative stress in a group of PTC patients compared to a group of BTG patients (Erdamar et al., 2010; Ramli et al., 2017), suggesting the role of oxidative stress in PTC tumourigenesis. Furthermore, differences in gene alteration patterns had been detected between BTG and PTC patients (Eng et al., 2023), providing insights into the molecular mechanisms underlying the benign-to-malignant transformation. However, studies evaluating and comparing oxidative stress and antioxidant capacity between benign and malignant thyroid lesions within the same patient are rarely performed. In this study, a comparison of antioxidant status, DNA repair capacity, and molecular alterations were conducted in benign and malignant lesions from the same individuals. The aim was to support earlier findings and to provide further insight into the mechanisms that drive the progression from benign to malignant thyroid nodules.

A significantly higher ROS level was detected in PTC lesions, possibly contributed by H_2_O_2_, an important component in thyroid hormone biosynthesis. Antioxidative response in the PTC lesions, evaluated through ABTS radical scavenging activity, FRAP analysis, GPx and SOD enzymatic activities, showed an upregulated trend, although not statistically significant. This may imply insufficient antioxidative power to counteract the high ROS accumulation, leading to oxidative stress. When the genes encoding GPx and SOD were evaluated through qRT-PCR, their expression was significantly higher in the malignant lesions compared to that in the benign lesions. This suggests that the high ROS level likely regulates both GPx and SOD at the gene transcription level instead of at the enzyme activity level.

*GPX1* encodes for glutathione peroxidase 1 (GPx1), which is the most abundant GPx isoform in the cytoplasm. It catalyses the cleavage of H_2_O_2_ to H_2_O in the presence of glutathione. Overexpression of the *GPX1* gene has been widely reported in human cancers (Wei et al., 2020). Similarly, upregulated *GPX1* expression has also been reported in thyroid tumours (Arczewska et al., 2020), although decreased GPx1 expression was observed through protein expression analyses (Metere et al., 2018; Muzza et al., 2022). SOD1 and SOD2 are isoforms of superoxide dismutase (SOD), which catalyse the dismutation of superoxide anion (O_2_^−^) to H_2_O_2_ and its subsequent conversion to H_2_O by other enzymes such as catalase, glutathione peroxidase and peroxiredoxins (Song et al., 2007). The expression of *SOD1* in our study showed no significant difference between the benign and malignant lesions, suggesting that the cytoplasmic O_2_^−^ level is similar between the two lesions. However, SOD2, located in the mitochondria, exhibited a significantly higher transcript level in the PTC lesions. The higher *SOD2* gene expression might indicate a high mitochondrial O_2_^−^ level, necessitating higher activity of SOD to eliminate it. Bidirectional H_2_O_2_ flux across the mitochondrial matrix and the cytosol has been reported (de Cubas et al., 2021). Under normal circumstances, mitochondrial H_2_O_2_ entering the cytosol is scavenged by peroxiredoxins to maintain cytosolic oxidative balance. Interestingly, the expression level of *PRDX1* in the present study was significantly lower in PTC lesions, which might suggest cellular impairment in scavenging the peroxides leaked from the mitochondria. A previous study by Nicolussi et al., 2013 also demonstrated reduced *PRDX1* expression in PTC compared to normal tissues, multinodular goitre and follicular neoplasms. The *PRDX6* expression level was also shown to be repressed in PTC patients in their study compared to patients with other thyroid neoplasms (Nicolussi et al., 2014). Additionally, a significantly lower *CAT* gene expression level was also detected in the PTC lesions in this study. Catalase (CAT) has a rate constant of approximately 10^7^ M/s, enabling the efficient conversion of H_2_O_2_ (Kurutas, 2016). A significant lower *CAT* mRNA expression was reported in thyroid tumours compared to the healthy tissues (Hasegawa et al., 2003). Thus, due to the reduced of catalase expression, GPx might not be sufficient in effectively removing the surfeit ROS in thyrocytes.

Oxidative imbalance induced by surfeit ROS production can lead to oxidative damage in biological macromolecules such as nucleic acids, proteins, and lipids. Although H_2_O_2_ is practically inert towards nucleic acids, the highly reactive hydroxyl radicals (·OH) produced during the Fenton reaction between H_2_O_2_ and transition metal ions, can cause oxidative DNA damage (Krohn et al., 2007). In DNA, guanine (G) is highly susceptible to oxidative damage due to its low ionisation potential (Steenken and Jovanovic, 1997; Margolin et al., 2006; Cadet et al., 2014). Two common 8-hydroxylated guanine derivatives, 8-oxoguanine (8-oxoG) and 8-hydroxy-2’-deoxyguanosine (8-OHdG), are products of cellular repair of oxidised guanine lesions (Arnett et al., 2005). The frequency of point mutations in DNA has been associated with its 8-oxoG content (Kuchino et al., 1987; Ribeiro et al., 1994). DNA damage coupled with aberrant DNA repair-related pathways, has been suggested to initiate thyroid tumourigenesis and play a crucial role in PTC progression from BTG (Ameziane El Hassani et al., 2019; Eng et al., 2023). *OGG1* codes for 8-oxoguanine glycosylase (OGG1), which participates in the BER pathway for the mutagenic 8-oxoG (Bruner et al., 2000; Izumi et al., 2003; Banerjee et al., 2005). In this study, a significantly higher expression of the *OGG1* gene and its encoded protein was observed in the malignant lesions compared to the benign lesions, indicating increased repair activity. Oxidative stress may have contributed towards DNA damage in the PTC lesions, necessitating higher OGG1 expression to remove oxidative DNA damage adducts. However, the level of 8-oxoG was not measured in this study, limiting the direct assessment of its correlation with OGG1 expression.

To further investigate whether the observed oxidative stress and the significantly higher expression of OGG1 in PTC lesions were reflected in genetic alterations, WES analysis was performed on biological triplicate of gDNA extracted from the benign and malignant lesions of the respective individuals. The Ti/Tv ratio has been utilised as a quality control parameter in high-throughput sequencing studies (Wang Gao et al., 2014). Typically, for human exome sequencing data, the Ti/Tv ratio is approximately 3 within protein coding regions and around 2 outside of exonic regions (Bainbridge et al., 2011). In our study, the average Ti/Tv ratio was determined to be 2.285, potentially influenced by the fact that WES also captures variants from non-exonic regions (Kasak et al., 2019). The VCF files generated from WES were subjected to CRAVAT analysis to identify the potential pathogenic driver mutations. The SNVs and InDels stratified by the CRAVAT tool were subjected to the filtering process to exclude synonymous variants/polymorphisms and mutations that might not be disease-related. Among the 169 genes that were found to harbour the potentially pathogenic variants, PTC lesions exhibited a higher number of those mutated genes (164 genes) compared to the BTG lesions (161 genes).

To unravel the underlying molecular events between benign and malignant lesions, pathway and process enrichment analysis were carried out. The genes with variants of high pathogenicity score in CRAVAT analysis were used as the input gene lists. The analysis had revealed different enriched GO biological processes between the BTG and PTC lesions. The four parental GO terms, namely “signaling”, “negative regulation of biological process”, “cellular process” and “developmental process” were found to be differentially enriched between BTG and PTC lesions. The development of PTC had been strongly linked to the oncogenic activation of signalling pathways that contributes to deviant cellular processes, such as cell proliferation, differentiation, apoptosis, and survival (Abdullah et al., 2019). A previous study had also demonstrated the gene alterations in signalling pathways to be an important event in PTC transformation (Eng et al., 2023). Among the biological processes that differentially enriched between BTG and PTC lesions, “regulation of MAPK cascade”, “positive regulation of ERK1 and ERK2 cascade” and “negative regulation of reactive oxygen species metabolic process” were the only terms that significantly enriched in PTC lesions.

Extracellular signal-regulated kinase 1/2 (ERK) belongs to MAPK family, transmitting extracellular signals to intracellular targets. Gene mutations that resulted in the oncogenic activation of MAPK signalling pathway have been found to be indispensable in the pathogenesis of many cancers, including *RET* chromosomal rearrangement, *BRAF* and *RAS* point mutations. Approximately 70% of PTC cases are found to have mutations involving one of the aforementioned genes. This study identified a total of 12 genes that affect the MAPK/ERK signalling pathway in PTC lesions, including *BRAF*, *MAP2K7*, *FGFR3*, *CD44*, *ABCA7*, *CD24*, *HIPK3*, *KSR1*, *SPAG9*, *HRH4*, *ERCC6* and *MAP3K9*. Of these genes, mutations in *BRAF*, *MAP2K7* and *CD24* were found exclusively in PTC lesions and were predicted to be pathogenic by CRAVAT variant filtering analysis. Although similarities were observed between the BTG and PTC lesions with regards to the mutated *FGFR3*, *CD44*, *ABCA7*, *CD24*, *HIPK3*, *KSR1*, *SPAG9*, *HRH4*, *ERCC6* and *MAP3K9*, the presence of these genes did not result in the enrichment of MAPK/ERK-related pathways in BTG lesions. Nonetheless, their presence may still be linked to the development of thyroid nodules. This observation raises the possibility that mutations in *BRAF*, *MAP2K7* and *CD24* are potentially contributing to the development and progression of PTC from BTG lesions. However, it is worth noting that the mutated *MAP3K9* gene, which belongs to the mitogen-activated protein kinase kinase (MAP3K) family and serves as an upstream regulator of the MAPK cascade, raises concerns, and requires further investigation.

*BRAF* encodes for a serine/threonine kinase, and the V600E mutation has been found to dramatically increase BRAF kinase activity. The rapid upregulation of the MAPK/ERK cascade results in cell proliferation, differentiation and tumourigenesis (Wan et al., 2004). Mutated *MAP3K9*, although not exclusively detected in PTC lesions, functions as an upstream regulator of MAPK signalling cascade. It phosphorylates and activates MAP2Ks, such as MAP2K4 and MAP2K7 (Stronach and Perrimon, 2002). Frequent somatic mutations in MAP3Ks, including *MAP3K5* and *MAP3K9*, were identified in melanoma patients (Stark et al., 2012). This study suggests that the presence of *MAP3K9* mutations in both BTG and PTC lesions, along with its role in the upstream regulation of MAPK cascade, may be implicated in the development of thyroid nodules. Conversely, mutations in downstream regulators, such as the *MAP2K7* gene, could potentially drive PTC progression. Mutations in the *RET*, *BRAF* and *RAS* genes are known to trigger the activation of the ERK1/ERK2 cascade. On the other hand, activation of MAP2K7 is frequently linked to the activation of the c-Jun N-terminal kinases (JNK) pathway, which is triggered in response to various cellular stressors (Cuevas and Schwab, 2017). This is not the first instance where changes in the molecular events of the *MAP2K7* gene have been associated with cancer (Hudson et al., 2018; Ray et al., 2020). Taken together, the higher ROS level observed in PTC lesions might serve as a source of cellular stressors, and mutations in the components of the MAPK/JNK cascade may trigger the activation of MAPK/JNK signalling pathways, leading to increased cell proliferation and the progression of PTC. Similarly, an increase in *CD24* expression has been demonstrated to positively regulate the MAPK cascade (Nakamura et al., 2017), but the association between *CD24* gene mutations and the PTC progression required further study.

The presence of an enriched biological process, namely “negative regulation of reactive oxygen species metabolic process”, provides further evidence for increased oxidative stress in PTC lesions. These findings emphasise the significance of exploring the relationship between oxidative stress and PTC, as well as developing targeted therapeutic strategies to address oxidative stress in PTC treatment. The H_2_O_2_ in thyrocytes is predominantly synthesised by dual oxidase 1 and 2 (DUOX1/2), members of the NADPH oxidase (NOX) family (Rigutto et al., 2009). Prolonged H_2_O_2_ exposure in thyrocytes has been shown to influence molecular events that interfere with MAPK and STAT3 pathways, mediated by the *BRAF* ^V600E^ mutation, impaired p53 functionality and *RET*/PTC1 chromosomal rearrangement (Ameziane-El-Hassani et al., 2010; Lee et al., 2016). *G6PD*, *SIRT2* and *BCR* were found to be related to the enriched ROS metabolism-related terms. The ROS scavenging activities of the major antioxidant systems, GPxs and PRDXs, are indispensable from the reduction capacity of NADPH (Hanschmann et al., 2013). Glucose 6-phosphate dehydrogenase (G6PD) is the rate-limiting enzyme of the pentose phosphate pathway (PPP), which is the major cellular source of NADPH (Nóbrega-Pereira et al., 2016). Thus, genetic alterations to G6PD can be hazardous due to the disruption in NADPH production. Sirtuin 2 (SIRT2) deacetylates several proteins in response to oxidative stress, such as PGC-1α and FOXO3a, modulates the upregulation of antioxidant enzymes expression and the reduction of ROS level (Wang et al., 2007; Krishnan et al., 2012). The study between *BCR* and oxidative stress is rarely performed, however, BCR-ABL cells were shown to have elevated ROS levels and oxidative DNA damage (Koptyra et al., 2008; Głowacki et al., 2021). It is also imperative to thoroughly explore the role of NADPH oxidase 4 (NOX4) in the excessive levels of ROS in PTC lesions, given NOX4’s association with various forms of cancer (Lin et al., 2017).

The CRAVAT filtering process identified four variants in PTC lesions, namely *BRAF*-rs113488022 (*BRAF* ^V600E^), *MAP2K7*-rs2145142862, *BCR*-rs372013175 and *CD24* p.Gln23fs mutation. *BRAF* ^V600E^ has been widely found in various types of neoplasms, including PTC (Nam et al., 2012; Silver et al., 2021; Eng et al., 2023), and its negative impact on BRAF kinase and the MAPK/ERK pathway is well-known (Cantwell-Dorris et al., 2011; Cohen et al., 2021). The variant *MAP2K7*-rs2145142862 is less commonly reported but is predicted to have a harmful effect based on *in silico* functional analysis. Previous studies have linked molecular changes in *MAP2K7* to cancer and an upregulation of the MAPK/JNK signalling pathway (Hudson et al., 2018; Ray et al., 2020). According to ClinVar, it has been recorded that the presence of the *BCR*-rs372013175 frameshift mutation might be associated with BCR-ABL positive chronic myelogenous leukemia (CML) (Allele ID: 1196252). The BCR-ABL fusion gene encodes for an activated ABL tyrosine kinase which can regulate varieties of cellular processes, such as cell growth, survival, and migration (Pendergast, 2002). The activation of the ABL tyrosine kinases was also detected in breast, colon, lung, kidney and melanomas (Greuber et al., 2013). Although this mutation has been reported to have a high prevalence in PTC patients, its role in PTC is yet to be determined (Qi et al., 2021). Whether there is an effect of *BCR*-rs372013175 in gene fusion process between *BCR* and *ABL*, remains largely unknown. The *CD24* gene is located on chromosome 6q21, but *CD24* homologous pseudogenes are also identified in chromosome 1, 15, 20 and Y (Fang et al., 2010). The *CD24* frameshift insertion variant identified in this study is located on chromosome Y, making it more difficult to predict its functional effect through *in silico* analysis. Further investigation is necessary to determine the potential role of *CD24* p.Gln23fs in the pathogenesis of PTC, as it was only identified in the PTC lesions.

In conclusion, our study revealed a heightened level of ROS and oxidative DNA damage in PTC lesions, as indicated by elevated OGG1 expression, compared to BTG lesions. These findings were supported by the gene alteration patterns in PTC lesions, which exhibited a significant enrichment of biological processes related to negative regulation of ROS metabolic process. Our study provides further evidence of the link between oxidative stress, DNA damage, and genetic changes associated with the transformation of BTG to PTC. The observed heightened ROS levels likely contribute to increased oxidative DNA damage, potentially initiating the BTG-to-PTC transformation through mutations in genes associated with the MAPK signalling pathway and stress-activated MAPK/JNK cascade. Further *in-vitro* investigations involving a larger sample size are needed to fully understand the impact of oxidative stress on the MAPK/JNK signalling pathway and its role in PTC progression.

## Data Availability

The VCF files utilised in this manuscript have been deposited on FigShare, with the DOI: 10.6084/m9.figshare.22225894. The files will be made public after the publication of this manuscript.

## References

[B1] Abdullah M. I. Junit S. M. Ng K. L. Jayapalan J. J. Karikalan B. Hashim O. H. (2019). Papillary thyroid cancer: genetic alterations and molecular biomarker investigations. Int. J. Med. Sci. 16 (3), 450–460. 10.7150/ijms.29935 30911279 PMC6428975

[B2] Ameziane El Hassani R. Buffet C. Leboulleux S. Dupuy C. (2019). Oxidative stress in thyroid carcinomas: biological and clinical significance. Endocrine-Related Cancer 26 (3), R131–R43. 10.1530/ERC-18-0476 30615595

[B3] Ameziane-El-Hassani R. Boufraqech M. Lagente-Chevallier O. Weyemi U. Talbot M. Métivier D. (2010). Role of H_2_O_2_ in *RET/PTC1* chromosomal rearrangement produced by ionizing radiation in human thyroid cells. Cancer Res. 70 (10), 4123–4132. 10.1158/0008-5472.CAN-09-4336 20424115

[B4] Arczewska K. D. Krasuska W. Stachurska A. Karpińska K. Sikorska J. Kiedrowski M. (2020). *hMTH1* and *GPX1* expression in human thyroid tissue is interrelated to prevent oxidative DNA damage. DNA Repair 95, 102954. 10.1016/j.dnarep.2020.102954 32877752

[B5] Arnett S. D. Osbourn D. M. Moore K. D. Vandaveer S. S. Lunte C. E. (2005). Determination of 8-oxoguanine and 8-hydroxy-2'-deoxyguanosine in the rat cerebral cortex using microdialysis sampling and capillary electrophoresis with electrochemical detection. J. Chromatogr. B 827 (1), 16–25. 10.1016/j.jchromb.2005.05.036 PMC2440692 15994136

[B6] Bainbridge M. N. Wang M. Wu Y. Newsham I. Muzny D. M. Jefferies J. L. (2011). Targeted enrichment beyond the consensus coding DNA sequence exome reveals exons with higher variant densities. Genome Biol. 12 (7), R68. 10.1186/gb-2011-12-7-r68 21787409 PMC3218830

[B7] Banerjee A. Yang W. Karplus M. Verdine G. L. (2005). Structure of a repair enzyme interrogating undamaged DNA elucidates recognition of damaged DNA. Nature 434 (7033), 612–618. 10.1038/nature03458 15800616

[B8] Bruner S. D. Norman D. P. G. Verdine G. L. (2000). Structural basis for recognition and repair of the endogenous mutagen 8-oxoguanine in DNA. Nature 403 (6772), 859–866. 10.1038/35002510 10706276

[B9] Cadet J. Wagner J. R. Shafirovich V. Geacintov N. E. (2014). One-electron oxidation reactions of purine and pyrimidine bases in cellular DNA. Int. J. Radiat. Biol. 90 (4), 423–432. 10.3109/09553002.2013.877176 24369822 PMC4047668

[B10] Cantwell-Dorris E. R. O'Leary J. J. Sheils O. M. (2011). BRAF^V600E^: implications for carcinogenesis and molecular therapy. Mol. Cancer Ther. 10 (3), 385–394. 10.1158/1535-7163.MCT-10-0799 21388974

[B11] Chatterjee N. Walker G. C. (2017). Mechanisms of DNA damage, repair, and mutagenesis. Environ. Mol. Mutagen. 58 (5), 235–263. 10.1002/em.22087 28485537 PMC5474181

[B12] Cohen R. Liu H. Fiskum J. Adams R. Chibaudel B. Maughan T. S. (2021). BRAFV600E mutation in first-line metastatic colorectal cancer: an analysis of individual patient data from the ARCAD database. JNCI J. Natl. Cancer Inst. 113 (10), 1386–1395. 10.1093/jnci/djab042 33734401 PMC7617278

[B13] Cuevas B. D. (2017). “Mitogen-activated protein kinase kinase kinases,” in Encyclopedia of cancer. Editor Schwab M. (Berlin, Heidelberg: Springer Berlin Heidelberg), 2872–2876.

[B14] de Cubas L. Pak V. V. Belousov V. V. Ayté J. Hidalgo E. (2021). The mitochondria-to-cytosol H_2_O_2_ gradient is caused by peroxiredoxin-dependent cytosolic scavenging. Antioxidants 10 (5), 731. 10.3390/antiox10050731 34066375 PMC8148214

[B15] Dean D. S. Gharib H. (2008). Epidemiology of thyroid nodules. Best Pract. Res. Clin. Endocrinol. Metabolism 22 (6), 901–911. 10.1016/j.beem.2008.09.019 19041821

[B16] Douville C. Carter H. Kim R. Niknafs N. Diekhans M. Stenson P. D. (2013). CRAVAT: cancer-related analysis of variants toolkit. Bioinformatics 29 (5), 647–648. 10.1093/bioinformatics/btt017 23325621 PMC3582272

[B17] Eng Z. H. Abdullah M. I. Ng K. L. Abdul Aziz A. Arba’ie N. H. Mat Rashid N. (2023). Whole-exome sequencing and bioinformatic analyses revealed differences in gene mutation profiles in papillary thyroid cancer patients with and without benign thyroid goitre background. Front. Endocrinol. 13, 13. 10.3389/fendo.2022.1039494 PMC9846740 36686473

[B18] Erdamar H. Çimen B. Gülcemal H. Saraymen R. Yerer B. Demirci H. (2010). Increased lipid peroxidation and impaired enzymatic antioxidant defense mechanism in thyroid tissue with multinodular goiter and papillary carcinoma. Clin. Biochem. 43 (7), 650–654. 10.1016/j.clinbiochem.2010.02.005 20171198

[B19] Fang X. Zheng P. Tang J. Liu Y. (2010). CD24: from A to Z. Cell. Mol. Immunol. 7 (2), 100–103. 10.1038/cmi.2009.119 20154703 PMC4001892

[B20] Głowacki S. Synowiec E. Szwed M. Toma M. Skorski T. Śliwiński T. (2021). Relationship between oxidative stress and imatinib resistance in model chronic myeloid leukemia cells. Biomol. [Internet] 11 (4), 610. 10.3390/biom11040610 PMC8074285 33924068

[B21] Greuber E. K. Smith-Pearson P. Wang J. Pendergast A. M. (2013). Role of ABL family kinases in cancer: from leukaemia to solid tumours. Nat. Rev. Cancer 13 (8), 559–571. 10.1038/nrc3563 23842646 PMC3935732

[B22] Hanschmann E-M. Godoy J. R. Berndt C. Hudemann C. Lillig C. H. (2013). Thioredoxins, glutaredoxins, and peroxiredoxins—molecular mechanisms and health significance: from cofactors to antioxidants to redox signaling. Antioxidants Redox Signal. 19 (13), 1539–1605. 10.1089/ars.2012.4599 PMC3797455 23397885

[B23] Hasegawa Y. Takano T. Miyauchi A. Matsuzuka F. Yoshida H. Kuma K. (2003). Decreased expression of catalase mRNA in thyroid anaplastic carcinoma. Jpn. J. Clin. Oncol. 33 (1), 6–9. 10.1093/jjco/hyg009 12604716

[B24] Hudson A. M. Stephenson N. L. Li C. Trotter E. Fletcher A. J. Katona G. (2018). Truncation and motif based pan-cancer analysis highlights novel tumor suppressing kinases. bioRxiv. 254813. 10.1126/scisignal.aan6776 PMC7984728 29666306

[B25] Izumi T. Wiederhold L. R. Roy G. Roy R. Jaiswal A. Bhakat K. K. (2003). Mammalian DNA base excision repair proteins: their interactions and role in repair of oxidative DNA damage. Toxicology 193 (1), 43–65. 10.1016/s0300-483x(03)00289-0 14599767

[B26] Kasak L. Rull K. Laan M. (2019). “Chapter 21 - genetics and genomics of recurrent pregnancy loss,” in Human reproductive and prenatal genetics. Editors Leung P. C. K. Qiao J. (United States: Academic Press), 463–494.

[B27] Koptyra M. Cramer K. Slupianek A. Richardson C. Skorski T. (2008). BCR/ABL promotes accumulation of chromosomal aberrations induced by oxidative and genotoxic stress. Leukemia 22 (10), 1969–1972. 10.1038/leu.2008.78 18401418 PMC3857962

[B28] Krishnan J. Danzer C. Simka T. Ukropec J. Walter K. M. Kumpf S. (2012). Dietary obesity-associated Hif1α activation in adipocytes restricts fatty acid oxidation and energy expenditure via suppression of the Sirt2-NAD^+^ system. Genes & Dev. 26 (3), 259–270. 10.1101/gad.180406.111 22302938 PMC3278893

[B29] Krohn K. Maier J. Paschke R. (2007). Mechanisms of disease: hydrogen peroxide, DNA damage and mutagenesis in the development of thyroid tumors. Nat. Clin. Pract. Endocrinol. Metabolism 3 (10), 713–720. 10.1038/ncpendmet0621 17893690

[B30] Kuchino Y. Mori F. Kasai H. Inoue H. Iwai S. Miura K. (1987). Misreading of DNA templates containing 8-hydroxydeoxyguanosine at the modified base and at adjacent residues. Nature 327 (6117), 77–79. 10.1038/327077a0 3574469

[B31] Kurutas E. B. (2016). The importance of antioxidants which play the role in cellular response against oxidative/nitrosative stress: current state. Nutr. J. 15 (1), 71. 10.1186/s12937-016-0186-5 27456681 PMC4960740

[B32] Lee C. C. Abdullah M. I. Mat Junit S. Ng K. L. Wong S. Y. Ramli N. S. F. (2016). Malignant transformation of benign thyroid nodule is caused by prolonged H_2_O_2_ insult that interfered with the STAT3 pathway? Int. J. Clin. Exp. Med. 9 (9), 18601–18617.

[B33] Lin X-L. Yang L. Fu S-W. Lin W-F. Gao Y-J. Chen H-Y. (2017). Overexpression of NOX4 predicts poor prognosis and promotes tumor progression in human colorectal cancer. Oncotarget 8 (20), 33586–33600. 10.18632/oncotarget.16829 28422720 PMC5464892

[B34] Margolin Y. Cloutier J-F. Shafirovich V. Geacintov N. E. Dedon P. C. (2006). Paradoxical hotspots for guanine oxidation by a chemical mediator of inflammation. Nat. Chem. Biol. 2 (7), 365–366. 10.1038/nchembio796 16751762

[B35] Metere A. Frezzotti F. Graves C. E. Vergine M. De Luca A. Pietraforte D. (2018). A possible role for selenoprotein glutathione peroxidase (GPx1) and thioredoxin reductases (TrxR1) in thyroid cancer: our experience in thyroid surgery. Cancer Cell Int. 18 (1), 7. 10.1186/s12935-018-0504-4 29371830 PMC5769232

[B36] Muzza M. Pogliaghi G. Colombo C. Carbone E. Cirello V. Palazzo S. (2022). Oxidative stress correlates with more aggressive features in thyroid cancer. Cancers 14 (23), 5857. 10.3390/cancers14235857 36497339 PMC9737385

[B37] Nakamura K. Terai Y. Tanabe A. Ono Y. J. Hayashi M. Maeda K. (2017). CD24 expression is a marker for predicting clinical outcome and regulates the epithelial-mesenchymal transition in ovarian cancer via both the Akt and ERK pathways. Oncol. Rep. 37 (6), 3189–3200. 10.3892/or.2017.5583 28440503 PMC5442399

[B38] Nam J. K. Jung C. K. Song B. J. Lim D. J. Chae B. J. Lee N. S. (2012). Is the BRAF^V600E^ mutation useful as a predictor of preoperative risk in papillary thyroid cancer? Am. J. Surg. 203 (4), 436–441. 10.1016/j.amjsurg.2011.02.013 21803329

[B39] Nicolussi A. D'Inzeo S. Mincione G. Buffone A. Di Marcantonio M. C. Cotellese R. (2014). PRDX1 and PRDX6 are repressed in papillary thyroid carcinomas via BRAF V600E-dependent and -independent mechanisms. Int. J. Oncol. 44 (2), 548–556. 10.3892/ijo.2013.2208 24316730

[B40] Nóbrega-Pereira S. Fernandez-Marcos P. J. Brioche T. Gomez-Cabrera M. C. Salvador-Pascual A. Flores J. M. (2016). G6PD protects from oxidative damage and improves healthspan in mice. Nat. Commun. 7 (1), 10894. 10.1038/ncomms10894 26976705 PMC4796314

[B41] Patel K. N. Yip L. Lubitz C. C. Grubbs E. G. Miller B. S. Shen W. (2020). The American association of endocrine surgeons guidelines for the definitive surgical management of thyroid disease in adults. Ann. Surg. 271 (3), E21–E93. 10.1097/SLA.0000000000003580 32079830

[B42] Paulson V. A. Rudzinski E. R. Hawkins D. S. (2019). Thyroid cancer in the pediatric population. Genes 10 (9), 723. 10.3390/genes10090723 31540418 PMC6771006

[B43] Pendergast A. M. (2002). The abl family kinases: mechanisms of regulation and signaling. Adv. Cancer Res. 85, 51–100. 10.1016/s0065-230x(02)85003-5 12374288

[B44] Qi T. Rong X. Feng Q. Sun H. Cao H. Yang Y. (2021). Somatic mutation profiling of papillary thyroid carcinomas by whole-exome sequencing and its relationship with clinical characteristics. Int. J. Med. Sci. 18 (12), 2532–2544. 10.7150/ijms.50916 34104084 PMC8176168

[B45] Ramli N. S. F. Mat Junit S. Leong N. K. Razali N. Jayapalan J. J. Abdul Aziz A. (2017). Analyses of antioxidant status and nucleotide alterations in genes encoding antioxidant enzymes in patients with benign and malignant thyroid disorders. PeerJ 5, e3365. 10.7717/peerj.3365 28584708 PMC5457668

[B46] Ray D. Yun Y. C. Idris M. Cheng S. Boot A. Iain T. B. H. (2020). A tumor-associated splice-isoform of *MAP2K7* drives dedifferentiation in MBNL1-low cancers via JNK activation. Proc. Natl. Acad. Sci. 117 (28), 16391–16400. 10.1073/pnas.2002499117 32601196 PMC7368273

[B47] Reczek C. R. Chandel N. S. (2017). The two faces of reactive oxygen species in cancer. Annu. Rev. Cancer Biol. 1 (1), 79–98. 10.1146/annurev-cancerbio-041916-065808 PMC11875389 40034143

[B48] Renuka I. V. Saila Bala G Fau - Aparna C. Aparna C Fau - Kumari R. Kumari R. Fau - Sumalatha K. Sumalatha K. (2012). The bethesda system for reporting thyroid cytopathology: interpretation and guidelines in surgical treatment. Indian J. Otolaryngology Heck Neck Surg. 64 (4), 305–311. 10.1007/s12070-011-0289-4 PMC3477437 24294568

[B49] Ribeiro D. T. De Oliveira R. C. Mascio P. D. Menck C. F. M. (1994). Singlet oxygen induces predominantly G to T transversions on a single-stranded shuttle vector replicated in monkey cells. Free Radic. Res. 21 (2), 75–83. 10.3109/10715769409056559 7921166

[B50] Rigutto S. Hoste C. Grasberger H. Milenkovic M. Communi D. Dumont J. E. (2009). Activation of dual oxidases Duox1 and Duox2: differential regulation mediated by camp-dependent protein kinase and protein kinase C-dependent phosphorylation. J. Biol. Chem. 284 (11), 6725–6734. 10.1074/jbc.M806893200 19144650 PMC2652333

[B51] Rosário P. W. S. Fagundes T. A. Borges M. A. R. Padrão E. L. Rezende L. L. Barroso Á. L. (2004). Completion thyroidectomy in patients with thyroid carcinoma initially submitted to lobectomy. Clin. Endocrinol. 61 (5), 652–653. 10.1111/j.1365-2265.2004.02148.x 15521973

[B52] Saad A. G. Kumar S. Ron E. Lubin J. H. Stanek J. Bove K. E. (2006). Proliferative activity of human thyroid cells in various age groups and its correlation with the risk of thyroid cancer after radiation exposure. J. Clin. Endocrinol. Metabolism 91 (7), 2672–2677. 10.1210/jc.2006-0417 16670159

[B53] Silver J. A. Bogatchenko M. Pusztaszeri M. Forest V-I. Hier M. P. Yang J. W. (2021). *BRAF V600E* mutation is associated with aggressive features in papillary thyroid carcinomas ≤ 1.5 cm. J. Otolaryngology - Head Neck Surg. 50 (1), 63. 10.1186/s40463-021-00543-9 PMC8572458 34742355

[B54] Song Y. Driessens N. Costa M. De Deken X. Detours V. Corvilain B. (2007). Roles of hydrogen peroxide in thyroid physiology and disease. J. Clin. Endocrinol. Metabolism 92 (10), 3764–3773. 10.1210/jc.2007-0660 17666482

[B55] Stark M. S. Woods S. L. Gartside M. G. Bonazzi V. F. Dutton-Regester K. Aoude L. G. (2012). Frequent somatic mutations in *MAP3K5* and *MAP3K9* in metastatic melanoma identified by exome sequencing. Nat. Genet. 44 (2), 165–169. 10.1038/ng.1041 PMC3267896 22197930

[B56] Steenken S. Jovanovic S. V. (1997). How easily oxidizable is DNA? One-electron reduction potentials of adenosine and guanosine radicals in aqueous solution. J. Am. Chem. Soc. 119 (3), 617–618. 10.1021/ja962255b

[B57] Stronach B. Perrimon N. (2002). Activation of the JNK pathway during dorsal closure in *Drosophila* requires the mixed lineage kinase, *slipper* . Genes & Dev. 16 (3), 377–387. 10.1101/gad.953002 11825878 PMC155330

[B58] Wan P. T. C. Garnett M. J. Roe S. M. Lee S. Niculescu-Duvaz D. Good V. M. (2004). Mechanism of activation of the RAF-ERK signaling pathway by oncogenic mutations of B-raf. Cell 116 (6), 855–867. 10.1016/s0092-8674(04)00215-6 15035987

[B59] Wang F. Nguyen M. Qin F. X-F. Tong Q. (2007). SIRT2 deacetylates FOXO3a in response to oxidative stress and caloric restriction. Aging Cell 6 (4), 505–514. 10.1111/j.1474-9726.2007.00304.x 17521387

[B60] Wang Gao T. Peng B. Leal Suzanne M. (2014). Variant association tools for quality control and analysis of large-scale sequence and genotyping array data. Am. J. Hum. Genet. 94 (5), 770–783. 10.1016/j.ajhg.2014.04.004 24791902 PMC4067555

[B61] Wei R. Qiu H. Xu J. Mo J. Liu Y. Gui Y. (2020). Expression and prognostic potential of GPX1 in human cancers based on data mining. Ann. Transl. Med. 8 (4), 124. 10.21037/atm.2020.02.36 32175417 PMC7049064

[B62] Zhang C. Wang X. Du J. Gu Z. Zhao Y. (2021). Reactive oxygen species-regulating strategies based on nanomaterials for disease treatment. Adv. Sci. 8 (3), 2002797. 10.1002/advs.202002797 PMC7856897 33552863

[B63] Zhou Y. Zhou B. Pache L. Chang M. Khodabakhshi A. H. Tanaseichuk O. (2019). Metascape provides a biologist-oriented resource for the analysis of systems-level datasets. Nat. Commun. 10 (1), 1523. 10.1038/s41467-019-09234-6 30944313 PMC6447622

